# Formulation and Evaluation of Chitosan-Chondroitin Sulphate Based Nasal Inserts for Zolmitriptan

**DOI:** 10.1155/2013/958465

**Published:** 2013-09-24

**Authors:** Kirandeep Kaur, Gurpreet Kaur

**Affiliations:** Department of Pharmaceutical Sciences and Drug Research, Punjabi University, Patiala, Punjab 147002, India

## Abstract

Bioadhesive nasal dosage forms are an attractive method for overcoming rapid mucociliary clearance transport in the nose and for delivering the drug directly to brain. The present study was designed to formulate chondroitin sulphate (CS) and chitosan (CH) nasal inserts employing zolmitriptan, an antimigraine drug. The interpolymer complexes (IPC) formed between –COO^−^ and –OSO_3_^−^ groups of CS and –NH_3_^+^ group of CH were characterized by infrared spectroscopy (IR), differential scanning analysis (DSC), and zeta potential studies. The unloaded and loaded nasal inserts were evaluated for water uptake studies, and bioadhesive strength studies, scanning electron microscopic studies (SEM). The *in vitro* drug release and *in situ* permeation studies were carried out on loaded nasal inserts. The DSC and IR studies confirmed the formation of a complex between the two polymers. The results indicated that the formulation F1 (CH : CS; 30 : 70) was demonstrating the highest bioadhesive strength and zeta potential. The presence of porous structure in the nasal inserts was confirmed by the SEM analysis. Further, *in vitro* and *in situ* release studies demonstrated that formulations F9 and F11 (drug : polymer; 1 : 10) were releasing 90% and 98% zolmitriptan over a period of 8 h. It can be concluded that nasal inserts formulated from chitosan-chondroitin sulphate (CH-CS) interpolymer complex (IPC) can be used for delivery of antimigraine drug to brain.

## 1. Introduction

Migraine is one of the most common headache disorders that affect 17-18% of the female and 6% of the male population. It is characterized by episodic unilateral headache attacks that are accompanied by nausea and/or photo- and phonophobia [1]. Current migraine treatment is divided into acute care and daily preventive medication. The acute treatment includes administration of simple analgesics like aspirin and NSAIDs; combination analgesics like caffeine with aspirin, acetaminophen, or caffeine; ergot alkaloids like ergotamine tartrate, dihydroergotamine, and triptans like sumatriptan, zolmitriptan; and naratriptan [2]. Migraine is frequently accompanied with severe nausea, poor gastrointestinal (GI) absorption, or vomiting; as a result oral administration of drugs becomes difficult [3]. The nasal route of administration offers an attractive alternative to the oral route for drug delivery. Intranasal drugs are transported along the olfactory sensory neurons to yield significant concentrations in the cerebrospinal fluids [4]. Intranasal route is noninvasive and painless, does not require sterility regulations, and is readily administered by the patient. However, the nasal cavity presents a number of limitations for drug absorption including enzymatic degradation, low intrinsic permeability for hydrophilic drugs, proteins, and peptides, and rapid mucociliary clearance. Therefore, in order to overcome these barriers a number of strategies can be applied by modifying three components: the drug, delivery carrier, and administration device [5]. Nasal inserts, a promising drug delivery system, are prepared by lyophilization of polymers. They consist of a sponge-like hydrophilic polymer matrix, in which the drug is embedded. They allow easy dosing with a high potential for systemic administration that helps prevent hepatic first pass metabolism. When the nasal insert comes into contact with the highly vascularized nasal mucosa, it absorbs water and swells. The polymer gel releases the active ingredient in a controlled manner [6]. Zolmitriptan is a second generation triptan prescribed for patients with migraine attacks, with or without aura and cluster headaches. It selectively acts on serotonin receptors and is very effective in reducing migraine symptoms, including pain, nausea, and photo- or phonophobia [7]. It is currently available as a conventional tablet, an oral disintegrating tablet, and a nasal spray. However, the bioavailability of nasal spray is very low due to rapid clearance of the drug from nasal cavity [8]. Therefore the emphasis now is to develop bioadhesive nasal delivery systems. Natural polysaccharides are generally regarded as safe (GRAS); they show interesting biological properties, including biocompatibility, biodegradability, and bioadhesion. A combination of polymers has been found to demonstrate better functional properties as compared to a single polymer. Further, tailor-made multiple polymers or interpolymer complexes have been exploited in the field of drug delivery to meet specific demands [9]. Therefore, in this study chitosan- (CH-) chondroitin sulphate (CS) interpolymer complexes (IPC) were employed for the formulation of bioadhesive nasal inserts. CH is a cationic polymer obtained by deacetylation of chitin, the polymer forming the exoskeleton of crustaceans. It has the capacity to open tight junctions and thus increases paracellular transport in the epithelial membrane, possesses nontoxicity, and low immunogenicity [10, 11]. Chondroitin sulphate (CS) is found in bone, cartilage, and connective tissue and composed of acetyl galactosamine and glucuronic acid residues alternately linked to each other by the *β*1-4 and *β*1-3 bonds, respectively. CS is an anionic mucopolysaccharide, which is able to form ionic complexes with positively charged substance [12]. CH-CS IPC were characterized by spectral and thermal analysis. The freeze-dried inserts were evaluated for their morphological characteristics, swelling studies, zeta potential, bioadhesive potential, and *in vitro *release and *in situ* permeation studies. 

## 2. Experimental

### 2.1. Materials

Chitosan was purchased from India Sea Foods, Cochin. Chondroitin sulphate was received as a gift sample from Panacea Biotech, Lalru. Zolmitriptan was purchased from Molekula Life Sciences Limited, Hyderabad. All other reagents and chemicals were of analytical grade and were used as such.

### 2.2. Preparation of CH-CS IPC

CH-CS IPC were prepared as reported by Luppi et al. [13]. CH and CS were dissolved separately in 25 mL of acetate buffer (pH 5.0) producing a 2% w/v of polymer solution. The CH solution was then added to CS solution in various molar ratios (30 : 70, 40 : 60, 50 : 50, 60 : 40, and 70 : 30; CH : CS) and stirred at room temperature for 24 h. The precipitates were separated by centrifugation (Remi Cooling Centrifuge, India) at 10,000 rpm for 10 min, washed with deionised water, and dried. The practical yield of the IPC was calculated employing the following equation:

(1) $$\begin{array}{c} \text{percent}\;\;\text{yield}\;\;\left(\%\right)=\frac{W_{0}}{W_{t}}\times 100, \end{array}$$

where *W*_0_ = weight of IPC obtained and *W*_*t*_ = total weight of polymers taken.

### 2.3. Characterization of IPC

#### 2.3.1. Attenuated Total Reflectance-Infrared (ATR-IR) Studies

The powdered polymers were characterized using ATR spectroscopy (ALPHA-E Bruker, Germany). The samples were scanned in the absorbance in the range from 4000 to 600 cm^−1^.

#### 2.3.2. Differential Scanning Calorimetric Studies (DSC)

DSC measurements were performed on powdered samples of pure CH and CS and their IPC in different ratios at a heating rate of 10°C/min (Mettler Toledo 812E, Switzerland) in the range from 30 to 400°C.

#### 2.3.3. Zeta Potential

Zeta potential (*ζ*) was measured using a Zetasizer (Malvern Instruments Ltd., Malvern, UK). The zeta potential measurements were performed by using an aqueous dip cell in an automatic mode. Samples were diluted in ultrapurified water and placed in a clear disposable zeta cell, with the cell position being adjusted.

#### 2.3.4. Viscosity Measurements of CH-CS IPC

The viscosity of the supernatants obtained after mixing solutions of CH with CS solutions in different ratios was determined on Brookfield viscometer spindle 00 (Brookfield viscometer LVDV1, Bruker, UK).

### 2.4. Preparation of Nasal Insert

The precipitates of CH and CS IPC were homogenized for 5 min, suspended again in deionised water, and finally freeze-dried (Allied Frost Macflow Engineering, India) in microcentrifuge tubes, obtaining unloaded solid inserts (Table 1). Mannitol was added to increase the mechanical strength of nasal inserts in the ratio of complex/mannitol of 9 : 1; w/w. Drug loaded nasal inserts were prepared by adding zolmitriptan in the doses of 2.5 mg and 5 mg to 50 mg (1 : 20 and 1 : 10 ratios, resp.) of different complex/mannitol mixtures (9 : 1; w/w) obtaining ten different inserts (Table 2). The unloaded inserts were evaluated for the water uptake studies and bioadhesive strength studies and the loaded inserts were evaluated for *in vitro* drug release and *in situ* permeation studies.

### 2.5. Evaluation of Nasal Inserts

#### 2.5.1. Water Uptake Studies

Accurately weighed unloaded nasal inserts (50 mg) containing different concentrations of polymers CH and CS were placed on filter paper soaked in phosphate buffer pH 5.5 and positioned on top of a sponge previously soaked in the hydration medium and placed in a petri plate filled with the same buffer to the height of 0.5 cm. The water uptake by the insert was calculated [13]. The swelling behavior of the sponge was calculated using the following equation:

(2) $$\begin{array}{c} P_{s}=\frac{\left(W_{s}-W_{i}\right)}{W_{i}}\times 100, \end{array}$$

where *P*_*s*_ is percent swelling, *W*_*s*_ is weight of the swollen insert at time “*t*,” and *W*_*i*_ is initial weight of the insert.

#### 2.5.2. Measurement of Bioadhesive Studies

The *in vitro *bioadhesive strength was evaluated using texture analyzer equipment equipped with a 50 Kg load cell (TA.XT Plus, Stable Micro Systems, UK). Excised nasal mucosa of an adult goat was used for the measurement of bioadhesive strength. It was obtained from a local slaughter house and placed in aerated saline solution at 4°C until used [14]. The nasal insert was mounted securely in place on a cylindrical probe (using double-sided adhesive tape) which was fixed to the mobile arm of the texture analyzer. The nasal mucosa was placed to the lower arm. The cylindrical probe with the membrane attached to its base was lowered at a speed of 0.5 mm/s at a force of 1 N for a contact time of 30 s. It was then withdrawn at a rate of 0.5 mm/s to a distance of 10 mm. The mucoadhesive performance of the samples was determined by measuring the resistance to the withdrawal of the probe (maximum detachment force) reflecting the mucoadhesion characterization of the polymeric formulations with nasal mucosa. At least three repetitions were obtained for each measurement.

#### 2.5.3. Scanning Electron Microscopy (SEM)

A very small amount of the sample was dropped on the sample holder with double-sided tape, extra powder was removed and coated for 70 s under an argon atmosphere with gold coating, and then the powdered samples on the SEM grid were allowed to air-dry for 10 min. Images were taken at different magnifications using SEM (Jeol JSM-6610 LV, USA) machine.

#### 2.5.4. Drug Content Uniformity

Nasal inserts were weighed individually and the drug was extracted in methanol. The drug content was determined by filtering the solution through 0.4 *μ*m membrane. The drug content was analyzed by measuring absorbance spectrophotometrically (Beckman DU640B spectrophotometer, USA) at 225 nm.

#### 2.5.5. *In Vitro* Drug Release Studies

*In vitro* drug release studies were carried out in phosphate buffer pH 5.5. using USP XXX-NF XXV (Apparatus-1-Basket type) dissolution apparatus (Tab Machines, India), where 450 mL of phosphate buffer pH 5.5 was used as dissolution media maintained at 37°C ± 0.5°C at 50 rpm. Drug loaded inserts (2.5 mg and 5 mg) were placed in the dissolution vessel. Five mL of aliquot samples was withdrawn at 1, 2, 3, 4, 5, 6, 7, and 8 h and replaced with fresh media. Samples were then filtered through a 0.45 *μ*m filter and analyzed spectrophotometrically at 225 nm. 

#### 2.5.6. *In Situ* Permeation Studies

*In situ *release study of loaded nasal insert was carried out by using Keshary-Chien Franz diffusion cell. The formulations containing 2.5 mg and 5 mg doses of zolmitriptan were placed in donor compartment and freshly prepared phosphate buffer pH 5.5 in the receptor compartment. The nasal mucosa was placed between donor and receptor compartment. The whole assembly was placed on the thermostatically controlled (37°C ± 0.5°C) magnetic stirrer. One mL of sample was withdrawn at predetermined time interval of 1 h for a period of 8 h and the same volume of fresh medium was replaced. The withdrawn samples were analyzed by UV spectrophotometer at 225 nm using a buffer as a blank. 

### 2.6. Statistical Analysis

In order to determine statistical significance, ANOVA was used and the differences were considered to be significant for values of *P* < 0.05.

## 3. Results and Discussion 

### 3.1. Characterization of IPC

**Figure 1:**
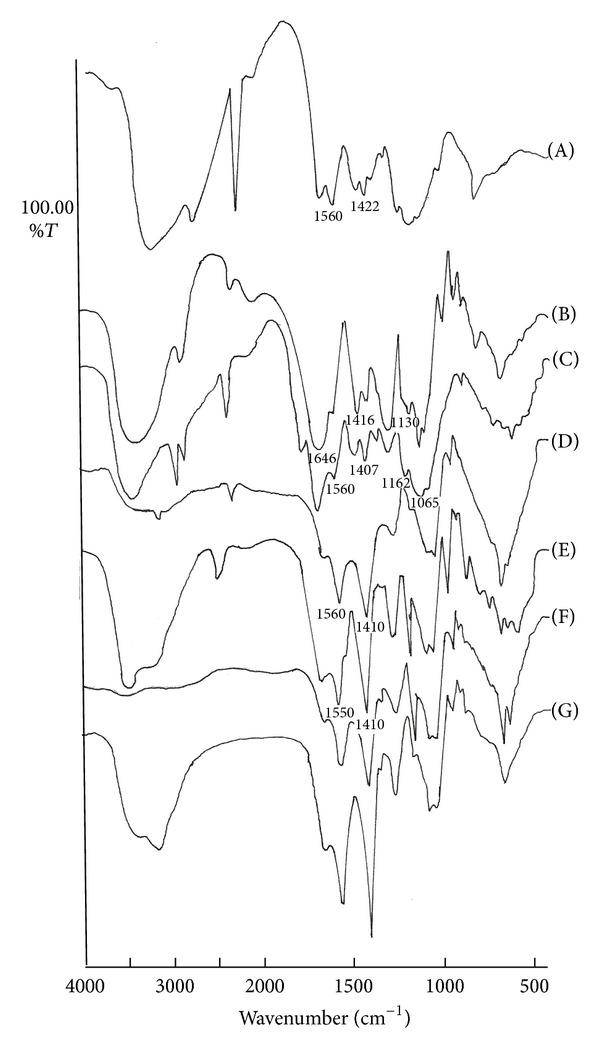
IR spectra of CH (A), CS (B), and IPCs in the ratio of CH : CS, (C) 70 : 30 (D) 60 : 40 (E) 50 : 50 (F) 40 : 60, and (G) 30 : 70.

**Figure 2:**
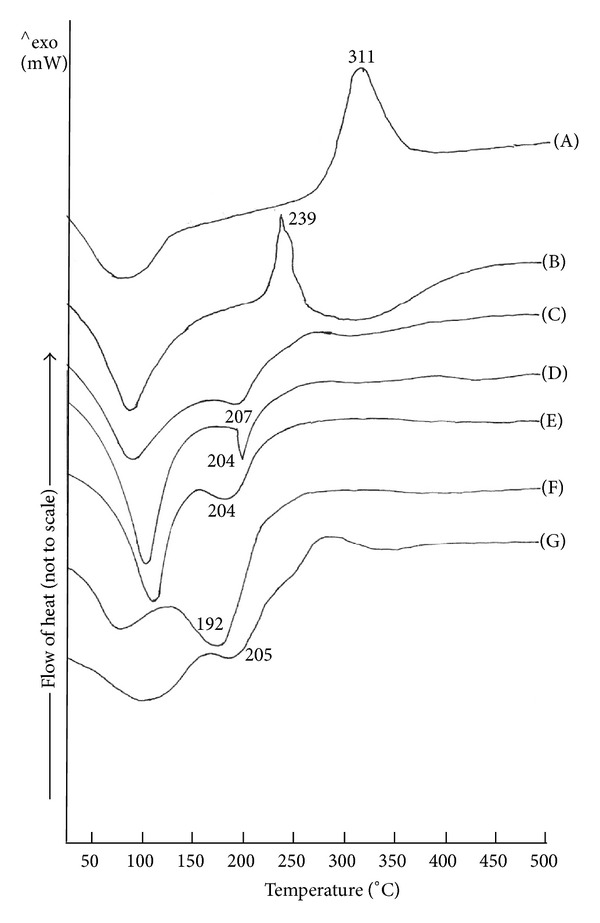
DSC thermograms of CH (A), CS (B), and their IPCs in the ratios of 70 : 30 (C), 60 : 40 (D), 50 : 50 (E), 40 : 60 (F), and 30 : 70 (G).

The percent yield of IPC obtained after complexation of CH and CS are depicted in Table 1. It was observed that maximum yields was obtained when CH and CS were present in the ratio of 50 : 50. The ATR-IR spectra of CH, CS and the IPC formed by interaction of various ratios of CH and CS showed characteristic bands (Figure 1). It is evident from Figure 1(a) that CH powder (85% deacetylation) exhibited a peak at 1560 cm^−1^ indicating presence of –NH_3_^+^ ions. The peak at 1422 cm^−1^ (Figure 1(a)), suggesting presence of –COO^−^ ions, could have arisen due to 15% acetylation of CH powder. The IR spectrum of CS is represented in Figure 1(b). The peaks observed at 1646 cm^−1^, 1416 cm^−1^, and 1130 cm^−1^ can be assigned, respectively, to –CONH_2_, –COO^−^, and −OSO_3_^−^ groups (Kemp, 1991). The CH-CS IPC displayed peaks in the ranges of 1400–1410 cm^−1^ and 1550–1560 cm^−1^ suggesting the formation of complex between –NH_3_^+^ and –COO^−^ groups of CH and CS, respectively (Figures 1(c), 1(d), 1(e), 1(f), and 1(g)). Further, the existence of a broad band at 1065 cm^−1^ with a shoulder at 1162 cm^−1^ in the IR spectrograph suggested the interaction between –NH_3_^+^ and −OSO_3_^−^ groups [15, 16].  Figure 2 depicts DSC thermograms of CH and CS which indicated one endothermic and one exothermic transition. It is evident from Figure 2(a) that CH powder exhibited one endothermic and one exothermic transition each at, respectively, 80.22°C and 311.30°C. The DSC thermogram of CS revealed one endotherm at 97°C and one exotherm at 239.69°C. The thermograms of IPC films containing any ratio of CH : CS exhibited the first endothermic transition with peak temperature ranging from 90 to 110°C. This was followed by a second endothermic transition with peak temperature ranging between 190 and 205°C (Figures 2(c)–2(g)). The first broad endotherm can be suggested to have arisen due to evaporation of water from the IPC. The second endothermic transition occurring in the temperature range of 190–205°C was found to differ in the Δ*H* value amongst films containing different ratios of CH : CS. This suggested that the second endothermic transition could be associated with the interaction between CH and CS during formation of IPC. 

Table 1 shows the viscosity of the supernatants obtained after mixing solutions of CH with CS solutions in different ratios. CS bears a negative charge owing to the presence of –COO^−^ and −OSO_3_^−^ groups. CH bears a net positive charge due to presence of −NH_3_^+^ groups. As a result, both polymers undergo spontaneous reaction when mixed together, resulting in formation of a solid mass. The viscosity of the supernatant obtained after mixing aqueous solutions of CH and CS was observed to increase as the proportion of CH in CH-CS mixtures increased (F4, F5). However, it was observed that the viscosity decreased to a value of 1.0 when CH and CS were admixed in equal proportions. Earlier studies carried out by Tapia et al. reported that the viscosity of supernatant obtained by admixing solutions of chitosan and carrageenan drops to almost unity when the proportion of chitosan was between 30 and 40% w/w. This was ascribed to the maximum interaction between chitosan and carrageenan from 30 : 70 to 40 : 60 ratios [17]. Hence, the present observation suggests maximum interaction between CH and CS at 50 : 50 ratio.

**Figure 3:**
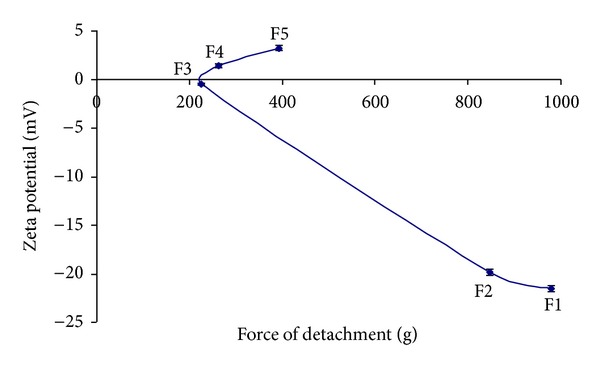
Relationship between force required for detachment and zeta potential.

The result of zeta potential values employing the dispersions of the CH and CS polymers alone and various ratios of the IPC is depicted in Figure 3. The IPC comprising 50 : 50 ratio of CH : CS showed almost negligible charges and this could be attributed to maximum interaction between –COO^−^ and −OSO_3_^−^ groups of CS with −NH_3_^+^ group of CH. The maximum value was of zeta potential was observed at a CH : CS ratio of 30 : 70. A slight positive value of zeta potential was reported for CH : CS; 60 : 40, 70 : 30 (Figure 3).

### 3.2. Evaluation of Nasal Inserts

#### 3.2.1. Water Uptake Studies

All the nasal inserts hydrated over a period of 8 h. The water uptake ability of all the nasal inserts is summarized in Table 1. The formulations comprising higher concentrations of CH (F4, F5; CH : CS 60 : 40, 70 : 30, resp.) showed higher water uptake ability (*P* < 0.05) as compared to inserts comprising low concentrations of CH (F1, F2; CH : CS 30 : 70, 40 : 60, resp.). Swelling is a function of the presence of ionized functional groups. The nasal insert prepared employing 50 : 50 ratio of CH : CS IPC (F3) was showing minimum water uptake since there was least number of ionized groups as result of maximum interaction [18]. The amine groups of CH are dissociated at pH 5.5; as a result high water uptake is observed in formulations comprising high concentrations of CH [19]. 

#### 3.2.2. Measurement of Bioadhesive Strength

Bioadhesive strength studies on investigational formulations were carried out using texture analyzer. The formulation F1 comprising 30 : 70 CH-CS IPC demonstrated the highest bioadhesive strength (maximum force required for detachment). These inserts were also depicting the highest value in zeta potential measurement indicating maximum number of ionized ions present in insert. The presence of free ionic groups in the polymeric matrix enables the polymer to interact with the functional groups present on the sialic acid residue of mucin. Further, polyanions (CS) have been reported to possess better bioadhesive strength as compared to polycations (CH) due to greater electrostatic interactions, hydrogen bonding, hydrophobic interaction, and interdiffusion because of similar chemical structures of the adhering components [20, 21]. Figure 3 depicts the relationship between force required for detachment of insert and zeta potential. An increase in the negative zeta potential has been reported to result in an increase in bioadhesion [22]. The IPC formed between CH and CS were demonstrating good bioadhesion, and this is likely to be due to the increased flexibility of the CH-CS IPC and polar functional groups. As a result of this, they are able to interact with the mucus chains and demonstrate good mucoadhesive strength, due to enhanced anchoring of the flexible chains with mucosa. Similar results have been reported by studies of chitosan/alginate hydrogels where it was found that the bioadhesive strength improved by tethering of long flexible chains to the particle surface. The resulting modified hydrogels exhibited increased mucoadhesive properties due to enhanced anchoring of the flexible chains with the mucosa [23].

#### 3.2.3. Scanning Electron Microscopy (SEM)

**Figure 4:**
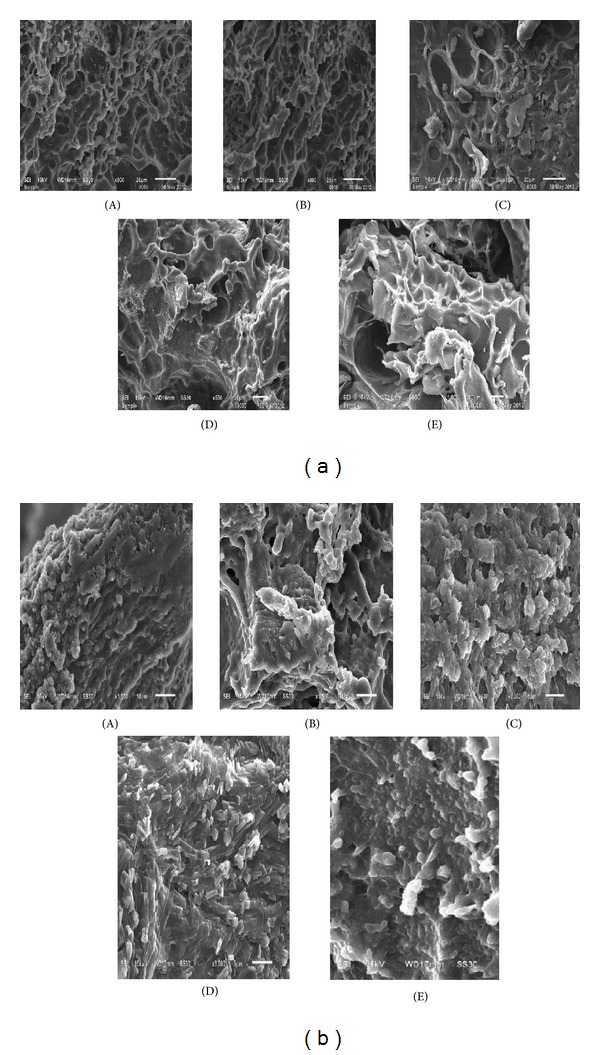
(a) SEM images of unloaded nasal insert: F1 (A), F2 (B), F3 (C), F4 (D), and F5 (E). (b) SEM images of loaded nasal inserts: F6 (A), F8 (B), F10 (C), F12 (D), and F14 (E).

Figures 4(a) and 4(b) show the morphology of the nasal inserts observed by scanning electron microscopy (SEM). The structure of the nasal inserts depends on the composition of CH-CS complexes. The process of freeze-drying is based on sublimation of the frozen water leading to the formation of pores or channels in the polymer. It was observed that CH (polycation) upon interaction with CS (polyanion) forms a physically cross-linked hydrogel. These hydrogels can retain a great amount of water at the interior. All the samples were characterized by a sponge-like structure [13]. Although the presence of higher amount of CH (F4, F5) provided the formation of a three-dimensional interconnected porous network structure with larger and more homogenous porosity with respect to higher amounts of CS, yet all the formulations seemed to possess suitable morphology as hydrogels for nasal administration due to their more evident sponge-like structure, which can provide great insert hydration and modulation of drug release in the nasal cavity. The SEM study showed that the presence of zolmitriptan in nasal insert produced a rough surface rather than a smooth one as unloaded samples and the porous structure tend to disappear.

#### 3.2.4. Drug Release Profiles

**Figure 5:**
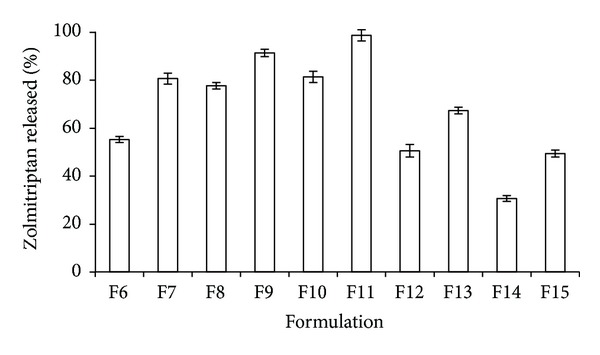
*In vitro* release of zolmitriptan from formulated nasal inserts.

**Figure 6:**
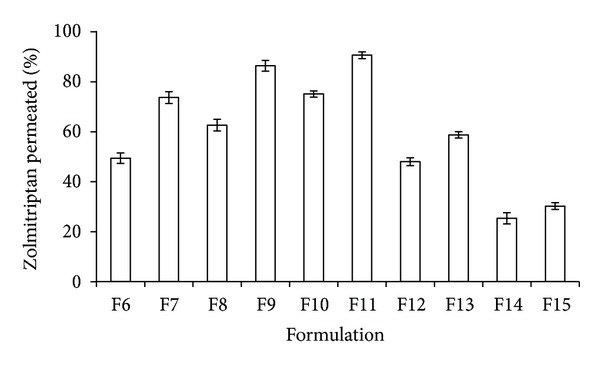
*In situ* permeation of zolmitriptan from formulated nasal inserts.

*In vitro *release studies were conducted using dissolution Apparatus-1-Basket type and phosphate buffer pH 5.5 was used as dissolution medium. The *in vitro* release profiles of zolmitriptan from drug loaded nasal inserts F6–F15 are depicted in Figure 5. Formulation F10 (CH : CS; 50 : 50) showed maximum drug release (98.56%) in 8 h. An increase in the CH concentration from 60% to 70% (formulations F12, F14; CH : CS 60 : 40, 70 : 30) resulted in a slow drug release (50% and 30%, resp., in 8 h). This slow drug release could be attributed to higher water uptake (swelling) of these formulations (3.98 and 4.52, resp.). Greater swelling of the polymers has been reported to result in an increase in the diffusional path length. Thus, the drug has to diffuse through the thick viscous gel layer formed outside the polymeric matrix hence resulting in slower drug release or release over prolonged period of time [24]. The nasal inserts were releasing statistically significant higher amounts of zolmitriptan from formulations containing drug-polymer ratio 1 : 10 as compared to 1 : 20 ratio of drug:polymer. *In situ* drug permeation studies also indicated that high amounts of zolmitriptan were permeating from formulations F9 and F11 (86% and 90%, resp.) as depicted in Figure 6. However, the force required for detachment was considerably high in F9 (845 ± 21 g) as compared to F11 (245 ± 15 g) thus indicating F9 to be a potential candidate for delivering zolmitriptan directly to brain.

## 4. Conclusion

This investigation demonstrated that CH-CS IPC can be employed for the formulation of nasal inserts for delivery of drugs to brain. The nasal inserts were displaying porous structure and good bioadhesive strength to overcome the nasal mucociliary clearance. Further intranasal absorption studies in animals are required to confirm delivery of zolmitriptan to brain.

## Figures and Tables

**Table 1:** Composition and properties of unloaded nasal inserts.

Formulation code	Composition (CH : CS)	Yield (%)	Viscosity (cP)	Water uptake
F1	30 : 70	33.68 ± 4.23	1.0 ± 0.0	2.88 ± 0.24
F2	40 : 60	52.45 ± 6.09	1.0 ± 0.0	2.07 ± 0.22
F3	50 : 50	55.22 ± 6.78	1.0 ± 0.0	1.65 ± 0.15
F4	60 : 40	49.09 ± 6.32	11.0 ± 1.0	3.98 ± 0.25
F5	70 : 30	35.35 ± 4.56	16.5 ± 1.5	4.52 ± 0.56

**Table 2:** Composition of drug loaded nasal inserts.

Formulation code	Composition (CH : CS)	Drug : polymer	Content uniformity (%)
F6	30 : 70	1 : 20	98.13 ± 0.77
F7	30 : 70	1 : 10	96.23 ± 0.80
F8	40 : 60	1 : 20	97.44 ± 0.70
F9	40 : 60	1 : 10	98.73 ± 0.83
F10	50 : 50	1 : 20	97.21 ± 1.25
F11	50 : 50	1 : 10	97.36 ± 0.96
F12	60 : 40	1 : 20	98.71 ± 1.56
F13	60 : 40	1 : 10	97.36 ± 1.45
F14	70 : 30	1 : 20	98.55 ± 0.98
F15	70 : 30	1 : 10	96.32 ± 1.65
